# Supplementary material for: Optimal PD-L1–high cutoff for association with overall survival in patients with urothelial cancer treated with durvalumab monotherapy
Source: PLoS One. 2020 Apr 27;15(4):e0231936. doi: 10.1371/journal.pone.0231936 (PMC7185603; doi:10.1371/journal.pone.0231936)
Supplement: S1 Table — (DOCX) [file pone.0231936.s001.docx]

## S1 Table. Full name of the ethics committee/institutional review board(s) that approved the study

| **Site Number** | **Investigator Name** | **Name of IRB/IEC** |
| --- | --- | --- |
| 1002501 | Maio, Michele | Comitato Etico per la Sperimentazione Clinica Dei Medicinali dell'A.O.U. Senese di Siena |
| 1053601 | Marshall, John | Georgetown University Institutional Review Board |
| 1056201 | Antonia, Scott | Liberty Institutional Review Board |
| 1062001 | Lebbé, Céleste | Association pour la Recherche le Traitement et l'Enseignement en Oncologie a Saint- Louis (ARTEOS) |
| 1062001 | Lebbé, Céleste | Hôpital Saint Antoine, Comité de Protection des Personnes Ile de France V |
| 1093501 | Hwu, Wen-Jen | Texas Oncology Austin Brain Tumor Center |
| 1094301 | Khleif, Samir | Georgia Regents University |
| 1245501 | Lutzky, Jose | Mount Sinai Hospital Research Ethics Board |
| 1322701 | Brahmer, Julie | Johns Hopkins Institutional Review Board |
| 1351901 | Segal, Neil | Memorial Sloan Kettering |
| 1371101 | Curiel, Tyler | University of Texas Health Science Center Committee for the Protection of Human Subjects |
| 1371501 | Schöffski, Patrick | Universitaire Ziekenhuizen Leuven Gasthuisberg |
| 1371701 | Massard, Christophe | Hôpital Saint Antoine, Comité de Protection des Personnes Ile de France V |
| 1372001 | Ascierto, Paolo Antonio | Comitato Etico Istituto Nazionale per lo Studio e la Cura dei Tumori Fondazione G. Pascale |
| 2000042 | Hamid, Omid | Western IRB |
| 2000043 | Gadgeel, Shirish | Western Institutional Review Board |
| 2000044 | Ou, Sai-Hong | University of California Davis Health System, Institutional Review Board |
| 2000044 | Ou, Sai-Hong | University of California, Irvine, Institutional Review Board |
| 2000045 | Butler, Marcus | University Health Network Research Ethics Board |
| 2000080 | Curigliano, Giuseppe | Comitato Etico Istituto Europeo di Oncologia e Centro Cardiologico Monzino |
| 2000089 | Sharma, Sunil | University of Utah Institutional Review Board |
| 2000090 | Zandberg, Dan | IRB-University of Maryland Medical Center |
| 2000108 | Gordon, Michael | Western IRB |
| 2000112 | O'Donnell, Peter | University of Chicago Institutional Review Board |
| 2000113 | Sanborn, Rachel | Providence Health and Services Institutional Review Board |
| 2000124 | Ott, Patrick | Dana-Farber Cancer Institute Institutional Review Board |
| 2000126 | Eder, Joseph | Yale University School of Medicine, Human Investigation Committee |
| 2000133 | Wainberg, Zev | University of California at Los Angeles Office for the Protection of Research Subjects (OPRS) |
| 2000134 | Gutierrez, Martin | Western IRB |
| 2000135 | Jamal, Rahima | Comité d'éthique de la recherche du CHUM |
| 2000136 | Nemunaitis, John | Mary Crowley Medical Research Center Institutional Review Board |
| 2000137 | Smith, David | University of Michigan Medical School Institutional Review Board |
| 2000166 | Sato, Takami | Thomas Jefferson University Institutional Review Board, Div. of Human Subjects Protection |
| 2000167 | Wakelee, Heather | Stanford University Institutional Review Board |
| 2000197 | Hoimes, Christopher | University Hospitals Case Medical Center, Institutional Review Board |
| 2000199 | Weiss, Jared | University of North Carolina, Office of Human Research Ethics, Biomedical Institutional Review Board |
| 2000199 | Weiss, Jared | Western Institutional Review Board |
| 2000206 | Jerusalem, Guy | CHU Sart Tilman-Comité d'Ethique Hospitalo-Facultaire Universitaire de Liège |
| 2000206 | Jerusalem, Guy | Universitair Ziekenhuis Leuven |
| 2000208 | Gianni, Luca | Comitato Etico dell'Ospedale San Raffaele |
| 2000209 | Keilholz, Ulrich | Landesamt für Gesundheit und Soziales Berlin, Geschäftsstelle der Ethik-Kommission des Landes Berlin |
| 2000210 | Jäger, Dirk | Ethikkommission der Universität Heidelberg |
| 2000211 | Cunningham, David | NRES Committee London - Surrey Borders |
| 2000213 | Middleton, Mark | NRES Committee London - Surrey Borders |
| 2000218 | Ciombor, Kristen | Western Institutional Review Board |
| 2000221 | Kim, Dong-Wan | Seoul National University Hospital Institutional Review Board |
| 2000228 | Kim, Sang-We | Asan Medical Center Institutional Review Board |
| 2000234 | Cunningham, David | NRES Committee London - Surrey Borders |
| 2000235 | Ahn, Myung-Ju | Samsung Medical Center Institutional Review Board |
| 2000410 | Goel, Sanjay | Biomedical Research Alliance of New York, Institutional Review Board |
| 2000437 | von Pawel, Joachim | Ethikkommission der Bayerischen Landesärztekammer |
| 2000438 | Romano, Gianpiero | Comitato Etico, Azienda Sanitaria Lecce |
| 2000439 | Rafii, Saeed | NRES Committee London - Surrey Borders |
| 2000675 | Shum, Merrill | Western Institutional Review Board |
| 2000677 | Mena, Raul | Providence Health and Services Institutional Review Board |
| 2000677 | Mena, Raul | Western Institutional Review Board |
| 2000678 | Powderly, II, John | Western Institutional Review Board |
| 2000679 | Shih, Kent | Western IRB |
| 2000680 | Smith, Pamela | Western IRB |
| 2000734 | Leach, Joseph | Quorum Review Institutional Review Board |
| 2000744 | Colon-Otero, Gerardo | Mayo Foundation Office for Human Research Protection |
| 2000747* | Bui, Lynne | Western IRB |
| 2001023 | Spira, Alexander | Western IRB |
| 2001024 | Blakely, Collin | Committee on Human Research |
| 2001061 | Nikolinakos, Petros | Western IRB |
| 2001062 | Chung, Ki-Young | Greenville Health System. |
| 2001062 | Chung, Ki-Young | Greenville Hospital System University Medical Center |
| 2001077 | Lee, Jong-Seok | Seoul National University Bundang Hospital Institutional Review Board |
| 2001090 | Sadjadian, Parvis | Ethik-Kommission der Ärztekammer Westfalen-Lippe und der Medizinischen Fakultät der Westfälischen Wilhelms-Universität Münster |
| 2001109 | Zylla, Dylan | Park Nicollet Institute Institutional Review Board for HealthPartners Institute |
| 2001115 | Goldschmidt, Jerome | Western IRB |
| 2001117 | Dasgupta, Anirudha | Western IRB |
| 2001118 | Gold, Philip | Western IRB |
| 2001185 | Goss, Glenwood | Ottawa Health Science Network Research Ethics Board |
| 2001190 | Braiteh, Fadi | Western IRB |
| 2002156* | Lin, Chia-Chi | Research Ethics Committee, National Taiwan University Hospital |
| 2002165 | Su, Wen-Pin | Institutional Review Board National Cheng Kung University Hospital |
| 2002166* | Ha, Hong Koo | Pusan University Hospital Institutional Review Board |
| 2002177 | Grimm, Marc-Oliver | Ethik-Kommission der Friedrich-Schiller-Universität Jena |
| 2002181 | Kang, Taek Won | Chonnam National University Hospital Institutional Review Board |
| 2002222 | Goeminne, Jean-Charles | Comité d’Ethique - Clinique Sainte-Elisabeth |
| 2002222 | Goeminne, Jean-Charles | Universitair Ziekenhuis Leuven |
| 2002252 | Rottey, Sylvie | Commissie voor Medische Ethiek - UZ Gent |
| 2002346 | Powles, Thomas | London – Surrey Borders Research Ethics Committee |
| 2002371 | Mao, Shifeng | Western IRB |
