# Supplementary material for: Optimal PD-L1–high cutoff for association with overall survival in patients with urothelial cancer treated with durvalumab monotherapy
Source: PLoS One. 2020 Apr 27;15(4):e0231936. doi: 10.1371/journal.pone.0231936 (PMC7185603; doi:10.1371/journal.pone.0231936)
Supplement: S2 Table — (DOCX) [file pone.0231936.s002.docx]

## S2 Table. Concordance index for overall survival based on cutoffs for PD-L1 expression.

| **Cutoff %** | Concordance - index  (C-index) |
| --- | --- |
| TC ≥ 1% | 0.505 |
| TC ≥ 10% | 0.474 |
| TC ≥ 25% | 0.514 |
| TC ≥ 50% | 0.496 |
| IC ≥ 1% | 0.614 |
| IC ≥ 10% | 0.606 |
| IC ≥ 25% | 0.698 |
| IC ≥ 50% | 0.711 |
| TC ≥ 1%/IC ≥ 1% | 0.589 |
| TC ≥ 10%/IC ≥ 25% | 0.572 |
| TC ≥ 25%/IC ≥ 25% | 0.664 |
| TC ≥ 50%/IC ≥ 25% | 0.675 |

IC, tumor-infiltrating immune cell; TC, tumor cell.
