# Supplementary figures and images for: Optimal PD-L1–high cutoff for association with overall survival in patients with urothelial cancer treated with durvalumab monotherapy
Source: PLoS One. 2020 Apr 27;15(4):e0231936. doi: 10.1371/journal.pone.0231936 (PMC7185603; doi:10.1371/journal.pone.0231936)

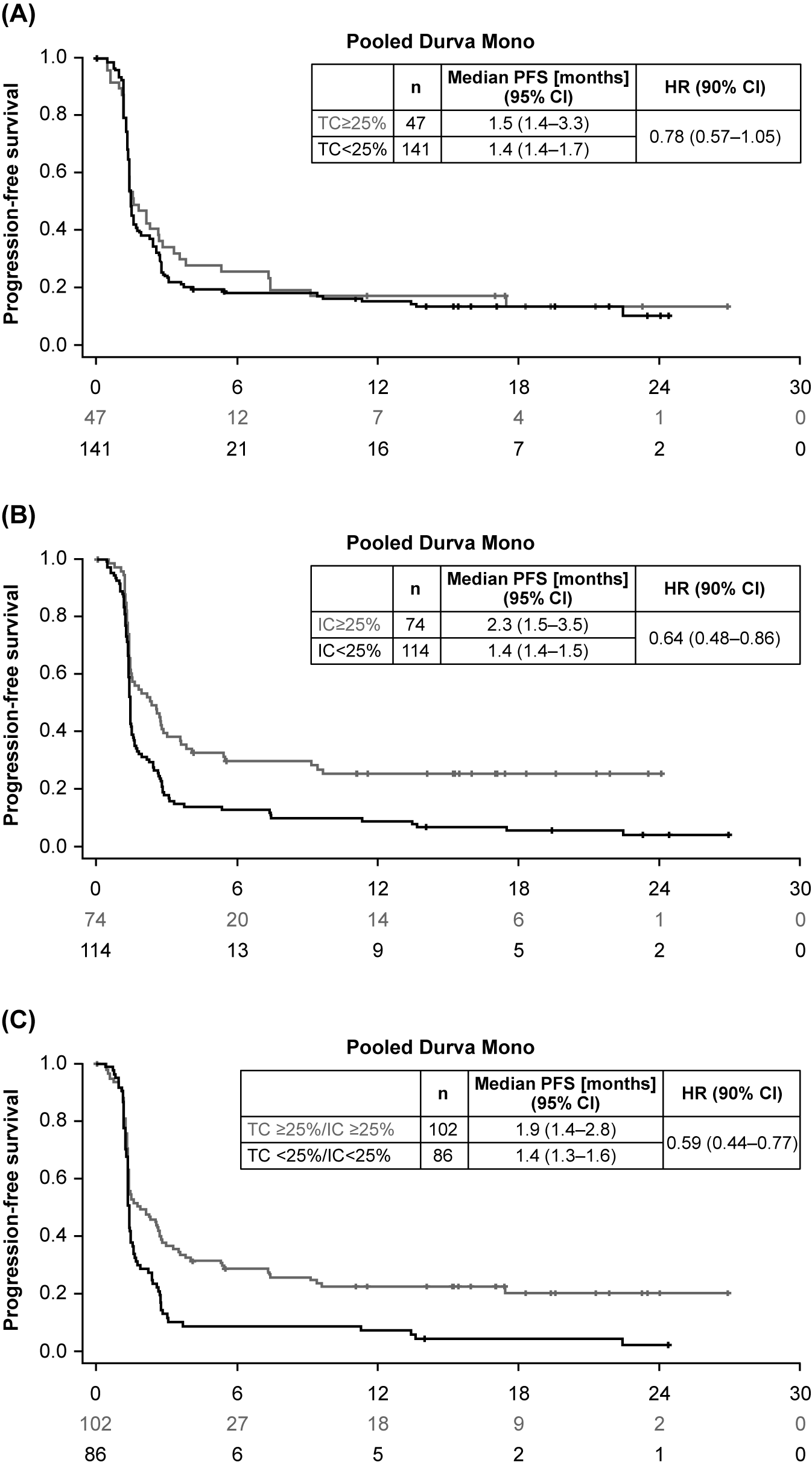

Supplement: S1 Fig — Tumor cells ≥ 25% (TC ≥ 25%; A), tumor-infiltrating immune cells ≥ 25% (IC ≥ 25%; B), and TC ≥ 25% or IC ≥ 25% (C). (TIF) [file pone.0231936.s003.tif]

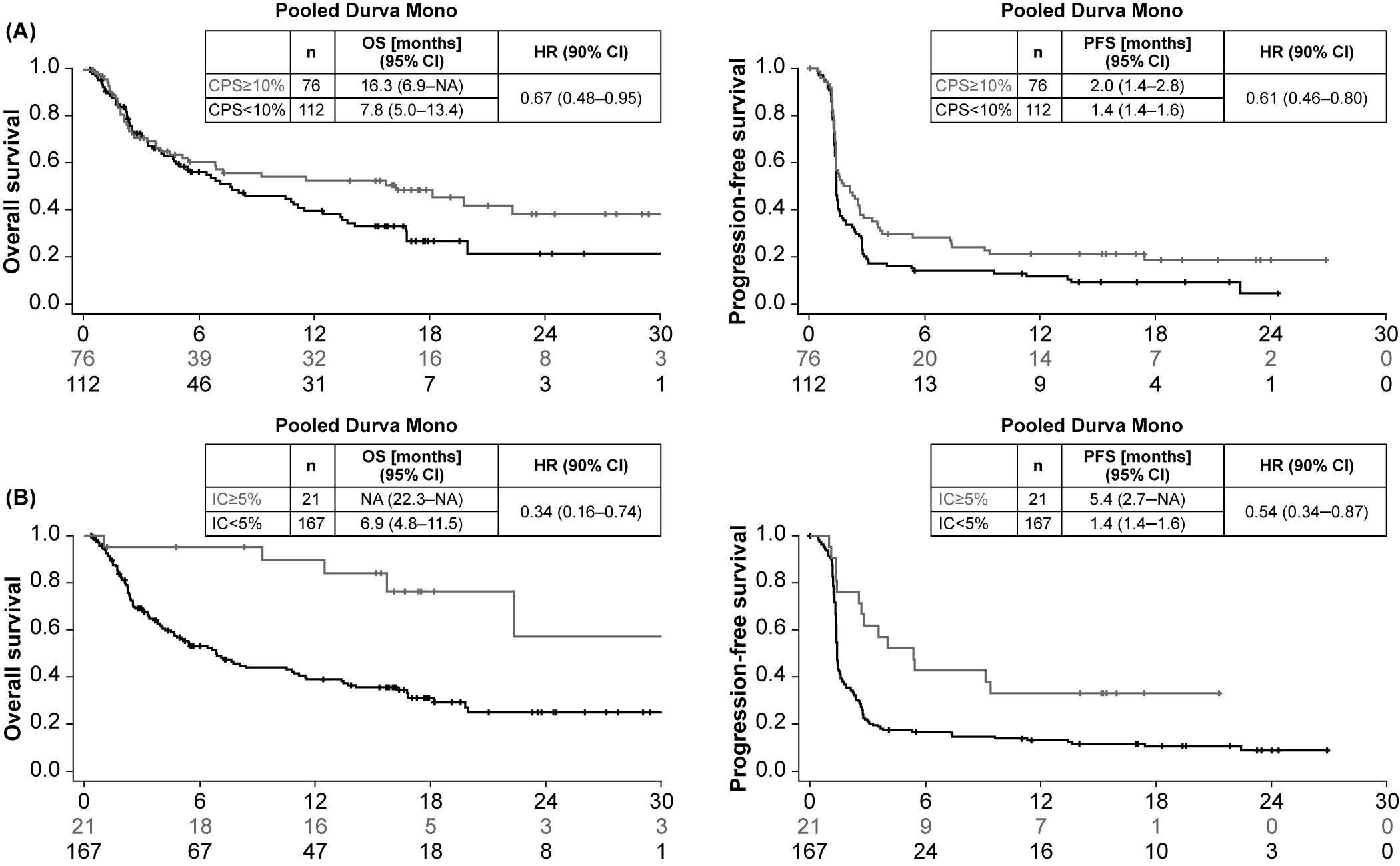

Supplement: S2 Fig — CPS ≥ 10, combined positive score defined as the percentage of PD-L1–expressing TC and IC relative to the total number of TC, with CPS ≥ 10 considered to be PD-L1–high; IC, tumor-infiltrating immune cells; IC ≥ 5%, ≥ 5% PD-L1 expression on IC; TC, tumor cells. (TIF) [file pone.0231936.s004.tif]
