# Supplementary material for: Optimal PD-L1–high cutoff for association with overall survival in patients with urothelial cancer treated with durvalumab monotherapy
Source: PLoS One. 2020 Apr 27;15(4):e0231936. doi: 10.1371/journal.pone.0231936 (PMC7185603; doi:10.1371/journal.pone.0231936)
Supplement: Attachment — Submitted filename: Response to reviewers_UC algorithm_23Aug19.docx [file pone.0231936.s005.docx]

Point by point response to reviewers for " Optimal PD-L1–high cutoff for association with overall survival in patients with urothelial cancer treated with durvalumab monotherapy " (PONE-D-19-18286)

| Reviewer comment | Response |
| --- | --- |
| 1. In line with the principles expressed in the Declaration of Helsinki, we expect all research involving human participants and/or medical data to have been approved by the authors' Institutional Review Board (IRB) or by equivalent ethics committee(s). If the need for ethical approval is waived, this should be formally confirmed by a suitable committee generally before the start of the study.  Therefore, before we can proceed further, please include in the Methods whether the study protocol was approved by an IRB or ethics committee and include the permit/approval number if so.  If approval was not sought, the ethics committee should formally confirm that ethical approval was not needed in this case. Please note that we do not accept retrospective ethics approval. (If approval is needed, this should have been done before the start of the study.) Please include a copy of the letter from the ethics committee as an "Other" file.  Some numerical values are expressed in too many decimal places, reflecting "superfluous precision". The SAMPL (Statistical Analyses and Methods in the Published Literature) guidelines on the EQUATOR Network repository (http://equator-network.org) say: "Report numbers . . . with an appropriate degree of precision. For ease of comprehension and simplicity, round to a reasonable extent." We recommend to adhere to the EASE guidelines state that in tables "numbers should be given in (sic) 2-3 effective digits, and APA Style requires consistently one or two decimal places. Please, replace three decimal places with two | The following paragraph has been added to the Methods section:  *Study 1108 was conducted according to the Declaration of Helsinki and approved by the independent ethics committee or institutional review board at each participating center, with written informed consent obtained from all patients; all data were anonymized prior to analysis.* *The inclusion of patients’ data in this post hoc analysis was allowed under the Study 1108 consent form. Study participants were not compensated. This study is registered with* [*ClinicalTrials.gov*](http://ClinicalTrials.gov) *number NCT01693562 and EudraCT number 2012-002206-52.*  We have uploaded a copy of the IRB approval for the lead investigator, Dr. Neil Segal of Memorial Sloan Kettering, as an example of the IRB approvals obtained for this multicenter study. The approval letter does not include a permit/approval number. Given the large volume of sites that participated in Study 1108, as well as the fact that the primary results have already been published, we have provided only 1 representative example IRB approval. |
| 2. In the ethics statement in the manuscript and in the online submission form, please provide additional information about the patient records used in this post-hoc analysis. Specifically, please ensure that you have discussed whether all data were fully anonymized before you accessed them and/or whether the IRB or ethics committee waived the requirement for informed consent.  If patients provided informed written consent to have data from their medical records used in research, please include this information. | Written informed consent was obtained from all patients for both the 1108 study and its related post hoc analyses and all data were anonymized prior to analysis. As noted in our response to comment #1, this information has been included in the Methods Section. |
| 3. In your Methods section, please provide additional information about the study population. Please ensure you have provided sufficient details to replicate the analyses such as:  a) the original recruitment date range (month and year),  b) a description of any inclusion/exclusion criteria that were applied, and  c) a table of relevant demographic details. | Inclusion/exclusion criteria and demographic details for Study 1108 have been published previously, and those previous publications are cited in our manuscript. We have updated the Methods Section to include the recruitment date range.  *The study design, inclusion and exclusion criteria, patient population, demographics, and clinical outcomes of the UC cohort enrolled in the single-arm, phase 1/2 1108 (NCT01693562) study have been reported elsewhere [7, 25].* *Study recruitment took place from August 2014 through June 2016. Data cutoff for the post hoc analysis reported here was October 2017.* |
| 4. Our staff editors have determined that your manuscript is likely within the scope of our Targeted Anticancer Therapies and Precision Medicine Call for Papers. This editorial initiative is headed by a team of Guest Editors for PLOS ONE: Andrew Cherniack, Anette Duensing, Steven Gray, Sunil Krishnan, Chandan Kumar-Sinha and Gayle Woloschak. The Collection will encompass a diverse range of research articles about the identification and classification of driver genes and somatic alterations, target and drug discovery, mechanisms of drug resistance, and early detection and screening. Additional information can be found on our announcement page: https://collections.plos.org/s/targeted-anticancer-therapies.  If you would like your manuscript to be considered for this collection, please let us know when you resubmit and we will ensure that your paper is treated as if you were responding to this call. If you would prefer to remove your manuscript from collection consideration, please specify this in the cover letter. | Thank you for alerting us to this opportunity; however, we would prefer not to be considered for this collection. Our cover letter has been updated accordingly. |
