# Supplementary material for: Optimal PD-L1–high cutoff for association with overall survival in patients with urothelial cancer treated with durvalumab monotherapy
Source: PLoS One. 2020 Apr 27;15(4):e0231936. doi: 10.1371/journal.pone.0231936 (PMC7185603; doi:10.1371/journal.pone.0231936)
Supplement: Attachment — Submitted filename: UC Algorithm_Response to reviewers_30Mar20_Submission.docx [file pone.0231936.s007.docx]

# Response to reviewers

30 March 2020

Dear Dr Hyunseok Kang,

My co-authors and I are pleased to submit a revised version of our original manuscript, titled “Optimal PD-L1 cutoff for association with overall survival in patients with urothelial cancer treated with durvalumab monotherapy,” for consideration of publication in PLOS ONE. (PONE-D-19-18286).

Please be aware that the page and line numbers provided below in the response to reviewers document match the tracked changes version of the updated manuscript, not the clean version of the manuscript.

The funding statement has been updated to:

The authors would like to thank the patients, their families and caregivers, and all investigators involved in the studies. The authors would also like to thank Marianne Ratcliffe (AstraZeneca) for her input. Medical writing support, which was in accordance with Good Publication Practice (GPP3) guidelines, was provided by Sherri Baber of Parexel (Hackensack, NJ).

The funder (AstraZeneca) provided support in the form of salaries for all authors, who contributed to the study design, data collection and analysis, decision to publish, or preparation of the manuscript. The specific roles of these authors are articulated in the ‘author contributions’ section.

The Competing Interests Statement

J. Ye, P. Mukhopadhyay, J. Walker, A. K. Gupta, M. C. Rebelatto, J. A. Williams are employees of AstraZeneca. M. Zajac, Y. Ben, X. Jin, J. Antal were employees of MedImmune/AstraZeneca at the time of manuscript development. These commercial affiliations do not alter our adherence to PLOS ONE policies on sharing data and materials.

Please find a point by point response to reviewers as requested below.

Kind regards

Jill Walker

Jill.Walker[@astrazeneca.com](mailto:@astrazeneca.com)

# Academic editor comments

1. Please ensure that your manuscript meets PLOS ONE's style requirements, including those for file naming. The PLOS ONE style templates can be found at <http://www.plosone.org/attachments/PLOSOne_formatting_sample_main_body.pdf> and <http://www.plosone.org/attachments/PLOSOne_formatting_sample_title_authors_affiliations.pdf>

**Response**:

- The manuscript has been copyedited in accordance with PLOS ONE’s style requirements

2. We note that you have indicated that data from this study are available upon request. PLOS only allows data to be available upon request if there are legal or ethical restrictions on sharing data publicly. For information on unacceptable data access restrictions, please see <http://journals.plos.org/plosone/s/data-availability#loc-unacceptable-data-access-restrictions>.

In your revised cover letter, please address the following prompts

a) If there are ethical or legal restrictions on sharing a de-identified data set, please explain them in detail (e.g., data contain potentially identifying or sensitive patient information) and who has imposed them (e.g., an ethics committee). Please also provide contact information for a data access committee, ethics committee, or other institutional body to which data requests may be sent.

b) If there are no restrictions, please upload the minimal anonymized data set necessary to replicate your study findings as either Supporting Information files or to a stable, public repository and provide us with the relevant URLs, DOIs, or accession numbers. Please see <http://www.bmj.com/content/340/bmj.c181.long> for guidelines on how to de-identify and prepare clinical data for publication. For a list of acceptable repositories, please see <http://journals.plos.org/plosone/s/data-availability#loc-recommended-repositories>.Some numerical values are expressed in too many decimal places, reflecting “superfluous precision”. The SAMPL (Statistical Analyses and Methods in the Published Literature) guidelines on the EQUATOR Network repository (http://equator-network.org) say: “Report numbers . . . with an appropriate degree of precision. For ease of comprehension and simplicity, round to a reasonable extent.” We recommend to adhere to the EASE guidelines state that in tables “numbers should be given in (sic) 2-3 effective digits, and APA Style requires consistently one or two decimal places. Please, replace three decimal places with two

**Response**

- The manuscript currently states that ‘Data underlying the findings described in this manuscript may be obtained in accordance with AstraZeneca’s data sharing policy described at: <https://astrazenecagrouptrials.pharmacm.com/ST/Submission/Disclosure>’
  - The statement has been moved to the end of the manuscript (*page 23, lines 430-432)*
  - We understand that the inclusion of this statement in the manuscript fulfils the criteria provided by PLoS One in their online data availability guidance (below):
    - When possible, we recommend authors deposit restricted data to a repository that allows for controlled data access. If this is not possible, directing data requests to a non-author institutional point of contact, such as a data access or ethics committee, helps guarantee long term stability and availability of data. Providing interested researchers with a durable point of contact ensures data will be accessible even if an author changes email addresses, institutions, or becomes unavailable to answer requests.

AstraZeneca support external requests for our clinical study data and documents. We are committed to sharing in the following ways:

- AstraZeneca Group of Companies [Data Request Portal](https://astrazenecagroup-dt.pharmacm.com/DT/Home) (leveraging our existing Trial Transparency System) allows researchers to submit a request to access de-identified patient level clinical data, aggregate clinical or genomics data (when available) and/or anonymized clinical study reports.
- An independent Scientific Review Board is available to review and approve requests. The Scientific Review Board will review requests that go back as far as 2009 through this process.
- All other requests for data prior to 2009 will continue to be reviewed by AstraZeneca on a case-by-case basis.
- All AstraZeneca Sponsored studies can be requested and will be considered for data and document sharing, including licensed and unlicensed products, when available.

In order for AstraZeneca to fulfill this commitment, AZ Guidelines for anonymizing patient level data are derived from the industry best practices today provided by organizations such as PhUSE, TransCelerate, and EMA Policy 0070 Guidance. Our guidelines are updated as knowledge, tools and best practices continue to evolve in order to achieve anonymization of clinical data. Anonymizing datasets enables us to fulfill our legal and ethical obligation to protect our patients whilst at the same time, utilizing patient’s data in progressing medical research to its full potential in the best interests of public health.

In the case of sharing anonymized patient level data, please note that the full dataset may not be shared in view of the following:

- Clinical informed consent for some countries prohibit secondary use of the data
- Patients may withdraw their consent for participation in the trial at any point
- Patient Privacy commitments require sponsors to manage the risks and ensure patients are not identified. AstraZeneca will measure the risk before sharing data and seek to ensure that less than three quasi identifiers are shared per patient.
- Other aspects might also be taken into consideration to protect patient privacy (e.g. review of rare clinical events where information is aggregated to a higher-level before sharing)
- For further information, the AstraZeneca Group of Companies – Data Request Portal (leveraging our existing Trial Transparency system) allows researchers to submit a request to access de‐identified patient level data. AstraZeneca shares clinical data through the SAS Multi‐Sponsor Environment, which is also an industry best practice solution. This tool allows researchers access to the data and use of a variety of analytical tools such as SAS, R, and other standards through a remote desktop solution. This environment allows AstraZeneca to fulfil our legal and ethical obligations as a good data steward, protecting the data from download and potential misuse.

3. Thank you for providing the following Funding Statement:

"This study was funded by AstraZeneca. Medical writing support, which was in accordance with Good Publication Practice (GPP3) guidelines, was provided by Sherri Baber of PAREXEL (Hackensack, NJ) and was funded by AstraZeneca. "

We note that one or more of the authors is affiliated with the funding organization, indicating the funder may have had some role in the design, data collection, analysis or preparation of your manuscript for publication; in other words, the funder played an indirect role through the participation of the co-authors.

If the funding organization did not play a role in the study design, data collection and analysis, decision to publish, or preparation of the manuscript and only provided financial support in the form of authors' salaries and/or research materials, please review your statements relating to the author contributions, and ensure you have specifically and accurately indicated the role(s) that these authors had in your study in the Author Contributions section of the online submission form. Please make any necessary amendments directly within this section of the online submission form.

Please also update your Funding Statement to include the following statement: “The funder provided support in the form of salaries for authors [insert relevant initials],but did not have any additional role in the study design, data collection and analysis, decision to publish, or preparation of the manuscript. The specific roles of these authors are articulated in the ‘author contributions’ section.”

If the funding organization did have an additional role, please state and explain that role within your Funding Statement.

**Response**

- The funding statement has been updated *(page 23, lines 434-443)*:

The authors would like to thank the patients, their families and caregivers, and all investigators involved in the studies. The authors would also like to thank Marianne Ratcliffe (AstraZeneca) for her input. Medical writing support, which was in accordance with Good Publication Practice (GPP3) guidelines, was provided by Sherri Baber of Parexel (Hackensack, NJ)

The funder (AstraZeneca) provided support in the form of salaries for all authors, who contributed to the study design, data collection and analysis, decision to publish, or preparation of the manuscript. The specific roles of these authors are articulated in the ‘author contributions’ section.

- The Author Contributions section of the online submission form includes the specific roles for each author *(page 24, lines 455-465)*:
  - **Conceptualization:** MZ, JW, JAW, PM
  - **Data Curation:** YB, JA, AKG
  - **Formal Analysis:** YE, XJ
  - **Investigation:** YB, JA, AKG, MCR, XJ
  - **Project Administration:** AKG, JW
  - **Supervision:** JW, PM
  - **Validation:** YE, PM, XJ
  - **Visualization: YE**
  - **Writing – Original Draft Preparation:** MZ, JAW, JW, YE
  - **Writing – Review & Editing:** All authors

4. Please also provide an updated Competing Interests Statement declaring this commercial affiliation along with any other relevant declarations relating to employment, consultancy, patents, products in development, or marketed products, etc.

**Response:**

The Competing Interests Statement has been updated (*page 24, lines 449-453*)

J. Ye, P. Mukhopadhyay, J. Walker, A. K. Gupta, M. C. Rebelatto, J. A. Williams are employees of AstraZeneca. M. Zajac, Y. Ben, X. Jin, J. Antal were employees of MedImmune/AstraZeneca at the time of manuscript development. These commercial affiliations do not alter our adherence to PLOS ONE policies on sharing data and materials.

5. Please amend the manuscript submission data (via Edit Submission) to include author Jill Walker.

**Response:**

- The manuscript submission data includes the author Jill Walker

6. Please amend your authorship list in your manuscript file to include author Lana Packham.

**Response:**

- Lana Packham is not an author of the manuscript and is an employee of Parexel, who provided medical writing and submission support

7. Thank you for stating the following in the Competing Interests section:

"M. Zajac, J. Ye, P. Mukhopadhyay, Y. Ben, and J. Walker are employees of AstraZeneca. X. Jin, J. Antal, A. K. Gupta, M. C. Rebelatto, and J. A.Williams are employees of MedImmune. "

We note that one or more of the authors are employed by a commercial company: AstraZeneca and MedImmune.

a. Please provide an amended Funding Statement declaring this commercial affiliation, as well as a statement regarding the Role of Funders in your study. If the funding organization did not play a role in the study design, data collection and analysis, decision to publish, or preparation of the manuscript and only provided financial support in the form of authors' salaries and/or research materials, please review your statements relating to the author contributions, and ensure you have specifically and accurately indicated the role(s) that these authors had in your study. You can update author roles in the Author Contributions section of the online submission form.

Please also include the following statement within your amended Funding Statement.

“The funder provided support in the form of salaries for authors [insert relevant initials], but did not have any additional role in the study design, data collection and analysis, decision to publish, or preparation of the manuscript. The specific roles of these authors are articulated in the ‘author contributions’ section.”

If your commercial affiliation did play a role in your study, please state and explain this role within your updated Funding Statement.

b. Please also provide an updated Competing Interests Statement declaring this commercial affiliation along with any other relevant declarations relating to employment, consultancy, patents, products in development, or marketed products, etc.

Within your Competing Interests Statement, please confirm that this commercial affiliation does not alter your adherence to all PLOS ONE policies on sharing data and materials by including the following statement: "This does not alter our adherence to  PLOS ONE policies on sharing data and materials.” (as detailed online in our guide for authors <http://journals.plos.org/plosone/s/competing-interests>) . If this adherence statement is not accurate and  there are restrictions on sharing of data and/or materials, please state these. Please note that we cannot proceed with consideration of your article until this information has been declared.

Please include both an updated Funding Statement and Competing Interests Statement in your cover letter. We will change the online submission form on your behalf.

Please know it is PLOS ONE policy for corresponding authors to declare, on behalf of all authors, all potential competing interests for the purposes of transparency. PLOS defines a competing interest as anything that interferes with, or could reasonably be perceived as interfering with, the full and objective presentation, peer review, editorial decision-making, or publication of research or non-research articles submitted to one of the journals. Competing interests can be financial or non-financial, professional, or personal. Competing interests can arise in relationship to an organization or another person. Please follow this link to our website for more details on competing interests: <http://journals.plos.org/plosone/s/competing-interests>

**Response:**

- a. The funding statement has been updated (*page 23, lines 433-443*)

The authors would like to thank the patients, their families and caregivers, and all investigators involved in the studies. The authors would also like to thank Marianne Ratcliffe (AstraZeneca) for her input. Medical writing support, which was in accordance with Good Publication Practice (GPP3) guidelines, was provided by Sherri Baber of Parexel (Hackensack, NJ).

The funder (AstraZeneca) provided support in the form of salaries for all authors, who contributed to the study design, data collection and analysis, decision to publish, or preparation of the manuscript. The specific roles of these authors are articulated in the ‘author contributions’ section.

- b. The Competing Interests Statement now states (*page 24, line 449-453*):

J. Ye, P. Mukhopadhyay, J. Walker, A. K. Gupta, M. C. Rebelatto, J. A. Williams are employees of AstraZeneca. M. Zajac, Y. Ben, X. Jin, J. Antal were employees of MedImmune/AstraZeneca at the time of manuscript development. These commercial affiliations do not alter our adherence to PLOS ONE policies on sharing data and materials.

7. We note that you have included the phrase “data not shown” in your manuscript. Unfortunately, this does not meet our data sharing requirements. PLOS does not permit references to inaccessible data. We require that authors provide all relevant data within the paper, Supporting Information files, or in an acceptable, public repository. Please add a citation to support this phrase or upload the data that corresponds with these findings to a stable repository (such as Figshare or Dryad) and provide and URLs, DOIs, or accession numbers that may be used to access these data. Or, if the data are not a core part of the research being presented in your study, we ask that you remove the phrase that refers to these data.

**Response:**

- The sentence referring to PFS curves has been removed from the manuscript.

8. Please upload a copy of Figure 5-6, to which you refer in your text on page 10-11. If the figure is no longer to be included as part of the submission please remove all reference to it within the text.

**Response:**

- Figures 5 and 6 have been added to the submission

9. Thank you for including your ethics statement: "This manuscript reports a post hoc analysis of a Phase I/II study that included human participants and/or tissue. As stated in the primary publication (published previously, this study was conducted according to the Declaration of Helsinki and was approved by the independent ethics committee or institutional review board at each of the 77 participating centers. Written informed consent to participate in the study was obtained from all patients. There was no separate consent regarding the post hoc analysis reported here."
a) Please amend your current ethics statement to include the full name of the ethics committee/institutional review board(s) that approved your specific study.

b) Once you have amended this/these statement(s) in the Methods section of the manuscript, please add the same text to the “Ethics Statement” field of the submission form (via “Edit Submission”).

For additional information about PLOS ONE ethical requirements for human subjects research, please refer to [http://journals.plos.org/plosone/s/submission-guidelines#loc-human-subjects-research](https://clicktime.symantec.com/33eCkTPSHwfmytP6n7sGFbK6H2?u=http%3A%2F%2Fjournals.plos.org%2Fplosone%2Fs%2Fsubmission-guidelines%23loc-human-subjects-research)

**Response:**

- The ethics statement has been updated in the manuscript and on the submission form to include reference to the number of sites involved (*page 7, line 141-142*). Due to the high number of sites (77) we have included a full list of the IRB approvals in the supporting files *(S1, p31-33).*
  - Study 1108 was conducted according to the Declaration of Helsinki and approved by the independent ethics committee or institutional review board at each of the 77 participating centers (full names of the ethics committees can be found in the supporting information, S1), with written informed consent obtained from all patients; all data were anonymized prior to analysis. The inclusion of patients’ data in this post hoc analysis was allowed under the Study 1108 consent form. Study participants were not compensated

# Reviewers comments

Reviewer 1

1. Labeling of the Figures should be re-done for consistency with the text. Especially Figure 2 and its sub-letters.

**Response**: Figures and text have been updated throughout to ensure alignment

2. Line 242: Objective Response Rate and this section.
   Authors should present the data in the following tablet format, to calculate PPV and NPV. The bar graphs in Figure 6 are inadequate when critiquing its role as a predictive biomarker of response.
   A good predictive biomarker has both positive predictive value and negative predictive value.

   Responders n =33 Non- responders n = 155
   PD-L1 High (cutoff __)n=__ ## #
   PD-L1 Low (cuffoff __)n=__ # ###

**Response**

- A table and related text [below], have been included to address the reviewers’ comment: *(page 15, lines 261-270)*

Cutoffs at IC ≥ 25%, TC ≥ 25/IC ≥ 25%, and IC ≥ 50% were associated with the highest positive predictive values (PPV; (28%, 28%, and 50%, respectively). The highest negative predictive values (NPV) were seen with IC ≥ 1% (95%), TC ≥ 1%/IC ≥ 1% (100%), and TC ≥ 25/IC ≥ 25% (94%). Optimizing for both NPV and PPV, TC ≥ 25%/IC ≥ 25% provided the optimal cutoff (Table 1).

**Table 1.** **Positive and negative predictive value of responders and non-responders in PD-L1 high and low negative patients.**

| Cutoff % | **PD-L1 high Responders**  **n** | **PD-L1 high Nonresponders**  **n** | **Positive predictive value**  **(95% CoI)** | **PD-L1 low/negative Responders**  **n** | **PD-L1 low/negative Nonresponders**  **n** | **Negative predictive value**  **(95% C I)** |
| --- | --- | --- | --- | --- | --- | --- |
| TC ≥ 1% | 25 | 91 | 0.22  (0.14-0.29) | 8 | 64 | 0.89  (0.82-0.96) |
| TC ≥ 10% | 15 | 62 | 0.20  (0.11-0.28) | 18 | 93 | 0.84  (0.77-0.91) |
| TC ≥ 25% | 11 | 36 | 0.23  (0.11-0.36) | 22 | 119 | 0.84  (0.78-0.90) |
| TC ≥ 50% | 7 | 29 | 0.19  (0.07-0.32) | 26 | 126 | 0.83  (0.77-0.89) |
| IC ≥ 1% | 30 | 104 | 0.22  (0.15-0.29) | 3 | 51 | 0.94  (0.88-0.01) |
| IC ≥ 10% | 29 | 98 | 0.23  (0.16-0.30) | 4 | 57 | 0.93  (0.87-1.00) |
| IC ≥ 25% | 21 | 53 | 0.28  (0.18-0.39) | 12 | 102 | 0.90  (0.84-0.95) |
| IC ≥ 50% | 14 | 31 | 0.31  (0.18-0.45) | 19 | 124 | 0.87  (0.81-0.92) |
| TC ≥ 1%/IC ≥ 1% | 33 | 125 | 0.21  (0.15-0.27) | 0 | 30 | 1  (1) |
| TC ≥ 10%/IC ≥ 25% | 28 | 92 | 0.23  (0.16-0.31) | 5 | 63 | 0.93  (0.86-0.99) |
| TC ≥ 25%/IC ≥ 25% | 28 | 74 | 0.28  (0.19-0.36) | 5 | 81 | 0.94  (0.89-0.99) |
| TC ≥ 50%/IC ≥ 25% | 26 | 70 | 0.27  (0.18-0.36) | 7 | 85 | 0.92  (0.87-0.98) |

3. Line 290 and 298: “IC> 5% cut-off..” does not appear to be consistent with the labeling of the Figures 1 and 6. Confirm that “CPS 10” and “IC2/3” on the Figures refer to CPS >10 and IC> 5%, respectively. If so, update the figure.

**Response**

- Figure labels have been updated to IC ≥ 5% and CPS ≥ 10 cutoffs

4. Line 334: Consider rephrasing this sentence: “TC>25%/IC>25% cutoff appeared to be optimal for excluding responders from the PD-L1-low population.” This cutoff separates the responders from non-responders.

**Response**

- Sentence has been updated to: ”Of particular note, the TC ≥ 25%/IC ≥ 25% cutoff appeared to be optimal for separating responders from nonresponders in the PD-L1–low population.” *(page 20, lines 350-352)*

Reviewer 2

1. C-statistics and C- index
   Basically, the authors find the optimal cutoff for the largest separation in the OS curves occurred. Not only minimize P-value and but also maximize the separation of OS curve, various cutoff values were categorized relative arbitrary. In order to overcome arbitrary cutoff, C statistics can be used. C-index for each cutoff level could measure the separability of OS curve likely with AUROC. It had better measure C-index

**Response**

- A table detailing the concordance-index has been included in the supplementary materials *(page 33)*
- A sentence referring to the concordance-index and table has been included *(page 10, lines 211-213)*
  - Concordance index (c-index), revealed that for the TCs, ≥ 25% had the highest c-index (0.514), and for IC, ≥ 25% and ≥ 50 % were highest (0.698 and 0.711, respectively) (S2 Table).

**S2 Table. Concordance index for overall survival based on cutoffs for PD-L1 expression.**

| **Cutoff %** | Concordance-index  (C-index) |
| --- | --- |
| TC ≥ 1% | 0.505 |
| TC ≥ 10% | 0.474 |
| TC ≥ 25% | 0.514 |
| TC ≥ 50% | 0.496 |
| IC ≥ 1% | 0.614 |
| IC ≥ 10% | 0.606 |
| IC ≥ 25% | 0.698 |
| IC ≥ 50% | 0.711 |
| TC ≥ 1%/IC ≥ 1% | 0.589 |
| TC ≥ 10%/IC ≥ 25% | 0.572 |
| TC ≥ 25%/IC ≥ 25% | 0.664 |
| TC ≥ 50%/IC ≥ 25% | 0.675 |

IC, tumor-infiltrating immune cell; TC, tumor cell
