# Supplementary material for: Optimal PD-L1–high cutoff for association with overall survival in patients with urothelial cancer treated with durvalumab monotherapy
Source: PLoS One. 2020 Apr 27;15(4):e0231936. doi: 10.1371/journal.pone.0231936 (PMC7185603; doi:10.1371/journal.pone.0231936)
Supplement: Attachment — Submitted filename: Reviewer comments.docx [file pone.0231936.s006.docx]

Overall well written. Robust survival data set.

Labeling of the Figures should be re-done for consistency with the text. Especially Figure 2 and its sub-letters.

**Line 242:** Objective Response Rate and this section.

Authors should present the data in the following tablet format, to calculate PPV and NPV. The bar graphs in Figure 6 are inadequate when critiquing its role as a predictive biomarker of response.

A good predictive biomarker has both positive predictive value and negative predictive value.

|  | Responders n =33 | Non- responders n = 155 |
| --- | --- | --- |
| PD-L1 High (cutoff __) n=__ | ## | # |
| PD-L1 Low (cuffoff __)n=__ | # | ### |

**Line 290 and 298:** “ IC≥ 5% cut-off..” does not appear to be consistent with the labeling of the Figures 1 and 6. Confirm that “CPS 10” and “IC2/3” on the Figures refer to CPS ≥10 and IC≥ 5%, respectively. If so, update the figure.

**Line 334**: Consider rephrasing this sentence: “TC≥25%/IC≥25% cutoff appeared to be optimal for *excluding* responders from the PD-L1-low population.” This cutoff separates the responders from non-responders.
